# Response to Letter to Editor: Comment on “Revisiting the Relationship Between Legal Awareness and Safe Abortion Utilization”

**DOI:** 10.1002/hsr2.73406

**Published:** 2026-10-18

**Authors:** Ololade Julius Baruwa, Emmanuel Banchani, Oluwaseyi Dolapo Somefun

**Affiliations:** ^1^ Department of Nursing and Public Health University of Fort Hare East London South Africa; ^2^ Department of Global and International Health University for Development Studies Ghana; ^3^ School of Public Health University of the Western Cape Cape Town South Africa

Editor,

We thank the authors of the Letter to the Editor for their comments on the article, “Awareness of Abortion Legality and Safe Abortion Uptake in Ghana: Assessing the Moderating Role of Education in a Cross‐Sectional Study” [1]. Their interest in the study and the opportunity to clarify the interpretation of the findings are appreciated. The concerns raised about the timing of legal awareness and recall bias are important when interpreting findings from cross‐sectional and retrospective data.

The first concern relates to whether women were aware of Ghana's abortion law before obtaining an abortion. This question is valid, but the 2017 Ghana Maternal Health Survey (GMHS) cannot establish this timing. Women included in the study reported an induced abortion that occurred within the 5 years before the survey, while awareness of abortion legality was measured at the time of the survey [1]. Therefore, it is not possible to determine whether women were aware of the law before their abortion.

This is important when interpreting the findings. Women who were aware of abortion legality had higher adjusted odds of reporting a safe abortion (aOR = 2.09; 95% CI: 1.42–3.08). However, this finding represents an association and should not be interpreted as evidence that legal awareness preceded or caused safe abortion uptake. This is a recognized limitation of cross‐sectional studies when the timing of the exposure and outcome cannot be clearly established [2, 3]. We also agree that reverse causation is possible. Women who obtained abortions from trained providers or recognized health facilities may have received information about Ghana's abortion law during their contact with the healthcare system. Therefore, some women may have become aware of the law during or after receiving abortion care rather than before seeking care. The GMHS did not measure women's legal awareness at the time of the abortion, making it impossible to determine which occurred first.

The second concern relates to recall bias and the possible misclassification of legal awareness. We agree that asking women to report abortion experiences that occurred within the previous 5 years may introduce recall error. Some women may not accurately remember details about the abortion method, provider, or place of care. In addition, abortion is a sensitive and often stigmatized issue, which may affect reporting [4, 5]. Legal awareness may also change over time. A woman may learn about Ghana's abortion law through education, media, community information, or contact with healthcare services after having an abortion. Therefore, legal awareness reported at the time of the survey may not reflect what she knew when the abortion occurred. This could result in misclassification of the exposure. However, the available data do not allow the extent of this misclassification to be determined or whether it increased or reduced the observed association.

These concerns are consistent with the limitation already acknowledged in the original article that the cross‐sectional design does not allow causal conclusions [1]. The comments further highlight two specific issues: possible reverse causation and changes in legal awareness between the abortion and the survey. The findings should therefore be interpreted as showing an association between legal awareness reported at the time of the 2017 GMHS and a reported safe abortion during the preceding 5 years. They do not establish that legal awareness preceded the abortion or caused women to obtain safer abortion care.

Nevertheless, the observed association remains important and warrants further investigation. Prospective or longitudinal studies would provide stronger evidence about the direction of this relationship. Future studies could assess women's knowledge of abortion law before abortion‐related decision‐making and then document subsequent care‐seeking, including the abortion method, provider, and place of care. Such studies could help determine whether legal awareness influences access to safe abortion services, whether contact with formal health services increases legal awareness, or whether both processes occur.

We thank the authors for their constructive comments. Their observations clarify the interpretation of the findings and highlight important areas for future research.

## Author Contributions

**Ololade Julius Baruwa:** conceptualization, writing – original draft, writing – review and editing. **Emmanuel Banchani:** conceptualization. **Oluwaseyi Dolapo Somefun:** conceptualization.

## Supporting information


Supporting File

## Data Availability

Data sharing is not applicable to this article as no datasets were generated or analyzed during the current study.
