# Supplementary material for: Response to Letter to Editor: Comment on “Revisiting the Relationship Between Legal Awareness and Safe Abortion Utilization”
Source: Health Sci Rep. 2026 Oct 18;9(10):e73406. doi: 10.1002/hsr2.73406 (PMC13678111; doi:10.1002/hsr2.73406)
Supplement: Supplementary file 1 [file HSR2-9-e73406-s001.docx]

Dear Editor,

We thank the reader for their careful assessment of our article, “Awareness of Abortion Legality and Safe Abortion Uptake in Ghana: Assessing the Moderating Role of Education in a Cross-Sectional Study,” and for highlighting important methodological considerations regarding temporality and recall. We appreciate the opportunity to clarify the interpretation of our findings.

**Comment 1: Temporal ambiguity and potential reverse causation**

The reader notes that awareness of abortion legality was measured at the time of the survey, whereas the abortion could have occurred at any point during the preceding five years. Consequently, it cannot be established that awareness preceded the abortion. The reader further notes that women who obtained abortion care from formal healthcare providers may have acquired information about the legal status of abortion during or after their interaction with the healthcare system, creating the possibility of reverse causation.

**Response:**
We agree with this observation. The 2017 Ghana Maternal Health Survey measured respondents' awareness of abortion legality at the time of interview and did not ascertain their awareness at the time the abortion occurred. Therefore, temporal ordering between legal awareness and abortion care-seeking cannot be established.

As acknowledged in our article, the cross-sectional design precludes causal inference. We agree that this limitation can be stated more specifically: the observed adjusted association between current legal awareness and reported safe abortion cannot establish that prior legal awareness influenced the decision to obtain a safe abortion. Reverse causation is plausible because interaction with formal healthcare providers could itself increase knowledge of the circumstances under which abortion is legally permitted in Ghana.

Accordingly, our estimate (aOR = 2.09; 95% CI: 1.42–3.08) should be interpreted as a cross-sectional association between reported legal awareness and safe abortion history rather than evidence that legal awareness caused or preceded safe abortion uptake. The association remains epidemiologically informative, but its direction and temporal sequence cannot be determined from these data.

We therefore agree with the reader that prospective or longitudinal research measuring legal awareness before abortion-related care-seeking would provide stronger evidence regarding temporality and direction of association.

**Comment 2: Five-year recall period, recall bias and possible exposure misclassification**

The reader observes that abortion experiences were self-reported for the five years preceding the survey and that respondents may have difficulty accurately recalling abortion methods, providers and facilities. The reader also notes that legal awareness may change over time and that awareness reported at the survey may not represent awareness at the time of the abortion, potentially resulting in exposure misclassification.

**Response:**
We agree that these are important measurement limitations. The GMHS collected retrospective information on induced abortions occurring within the five years preceding the survey, including information used to classify abortion safety. As with retrospective self-reported reproductive health data, these measures are potentially susceptible to recall error and reporting bias.

We also agree that the concern regarding legal awareness is particularly important. Awareness of abortion law is not necessarily time-invariant. A woman's knowledge could change between the abortion event and the survey through education, media exposure, community information, subsequent reproductive healthcare encounters, or the abortion-care experience itself. Consequently, current legal awareness cannot be assumed to represent awareness at the time the abortion decision was made.

Such temporal discordance may result in exposure misclassification. However, the available GMHS data do not permit us to determine the direction or magnitude of this potential misclassification and, consequently, whether it biased the observed association toward or away from the null.

Our original article acknowledged the potential for reporting bias associated with sensitive abortion information. We appreciate the reader's observation because it identifies more specifically the potential for recall error and temporal misclassification of legal awareness.

**Overall response**

We thank the reader for these constructive methodological observations. We agree that temporal ambiguity, possible reverse causation, recall error and exposure misclassification warrant explicit consideration when interpreting our findings. These limitations do not alter the reported statistical association, but they appropriately constrain its interpretation.

Our principal conclusion should therefore be understood as evidence that women who reported awareness of abortion legality at the time of the 2017 GMHS had higher adjusted odds of reporting an abortion classified as safe within the preceding five years. The findings should not be interpreted as demonstrating that legal awareness preceded, caused, or increased safe abortion uptake.

We further agree that prospective studies that measure legal awareness before abortion-related decision-making, alongside contemporaneous measures of abortion methods, providers and places of care, would provide stronger evidence regarding the temporal and potentially causal relationship between legal awareness and safe abortion care.

We appreciate the reader's contribution to the interpretation of our study.

Sincerely,

Ololade Julius Baruwa
On behalf of the authors
